# 1990s *Vibrio cholerae* Epidemic, Brazil

**DOI:** 10.3201/eid1101.040484

**Published:** 2005-01

**Authors:** Ana C.P. Vicente, Ana M. Coelho

**Affiliations:** * Institute Oswaldo Cruz, Rio de Janeiro, Brazil; † Federal University of Rio de Janeiro, Brazil

**Keywords:** letter, Vibrio cholerae, seventh pandemic, cholera, Brazil

**To the Editor:** We read with interest the letter by Sarkar et al. on new *Vibrio cholerae* phages (*1*). The description of new *V. cholerae* phages is a welcome tool for epidemiologic studies of this species. Our main concern about their work is the inaccurate picture that is presented of the cholera epidemic in Brazil. Some of the statements made in the final paragraphs are in disagreement with the official epidemiologic records and the characteristics of the *Vibrio* bacteria that occurred in Brazil during the 1990s epidemic (*2*).

In 1991, the seventh cholera pandemic reached South America by the Pacific coast, spreading to Brazil in the same year (*3*). In Brazil, the first cholera cases were reported in the Amazon region bordering Peru; within a few months a large number of cholera cases were recorded in states facing the Atlantic Ocean in the northeastern region (*2*). According to the official figures of the Brazilian Ministry of Health (*2*), 168,598 cases of cholera caused by a *V. cholerae* O1 El Tor strain occurred in Brazil from 1991 to 2001. Of these, 155,363 (92.1%) occurred in the northeastern area of the country, with 2,037 deaths. From 2001 to 2003, the number of confirmed cases was 4,756, 734, and 7, respectively.

Sarkar et al. (*1*) indicate that 60,000 cases occurred from 1991 to 2001 in Rio de Janeiro, a city localized in the southeastern region; the official records report only 349 cases. The statement that “since 1993, no cholera cases caused by O1 have been reported” is also perplexing. From 1994 to 2001, the official records report 68,583 cases of cholera in Brazil (51,324 of these in 1994, the second major year of cholera incidence). Are the authors suggesting that this number of cases was caused by non-O1 *V. cholerae*? The official records state that the cholera epidemic in Brazil was caused by an El Tor O1 strain (*4*,*5*).
